# Methylene Blue as a Photo-Redox Catalyst: The Development Synthesis of Tetrahydrobenzo[*b*]pyran Scaffolds via a Single-Electron Transfer/Energy Transfer

**DOI:** 10.3389/fchem.2022.934781

**Published:** 2022-07-18

**Authors:** Farzaneh Mohamadpour

**Affiliations:** School of Engineering, Apadana Institute of Higher Education, Shiraz, Iran

**Keywords:** methylene blue (MB^+^), photo-redox catalyst, renewable energy source, tetrahydrobenzo[b]pyran scaffolds, aqueous solvent, photochemical synthesis

## Abstract

In a green tandem reaction using aldehyde derivatives, malononitrile, and dimedone, a radical tandem Knoevenagel–Michael cyclocondensation reaction of tetrahydrobenzo[*b*]pyran scaffolds was developed. Using visible light as a sustainable energy source, methylene blue (MB^+^)-derived photo-excited state functions were employed in an aqueous solution as single-electron transfer (SET) and energy transfer catalysts. The range of yields is quite uniform (81–98%, average 92.18%), and the range of reaction time is very fast (2–7 min, average 3.7 min), and the point mentioned in the discussion is that the procedure tolerates a range of donating and withdrawing groups, while still giving very excellent yields. The reaction is fairly insensitive to the nature of the substituents. Research conducted in this project aims to develop a non-metallic cationic dye that is both inexpensive and widely available for more widespread use. In addition to energy efficiency and environmental friendliness, methylene blue also offers an excellent atom economy, time-saving features, and ease of use. As a result, a wide range of long-term chemical and environmental properties can be obtained. The turnover number and turnover frequency of tetrahydrobenzo[*b*]pyran scaffolds have been computed. Surprisingly, gram-scale cyclization is a possibility, implying that the technology may be applied in industries.

## Graphical Abstract

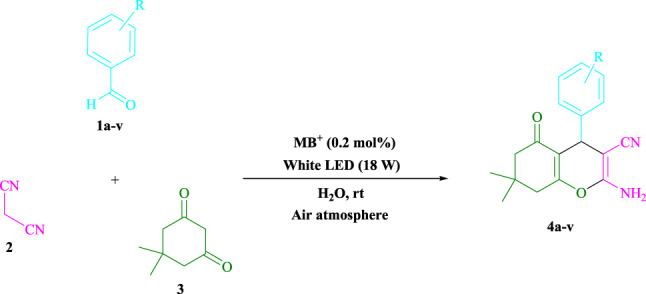

## 1 Introduction

**FIGURE 1:**
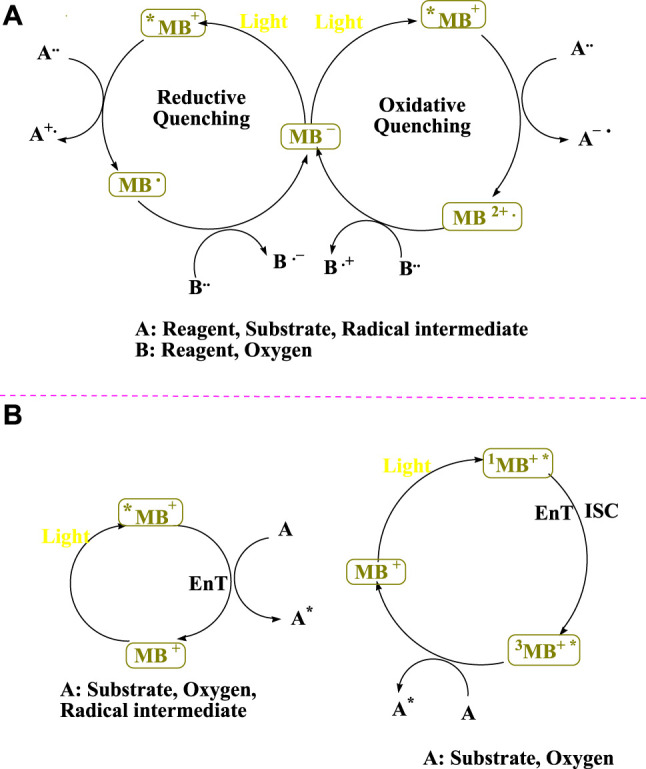
Photocatalytic cycles can be carried out with MB^+^ (Patel et al., 2021).

Photoredox catalysts have recently played an increasingly important role in the organic synthesis by forming C–C and C–heteroatom bonds via single electron transfer (SET) and photo-induced electron transfer (PET). From small-scale to large-scale, they are required for a variety of treatments. Technological advances have led to the development of flow reactors (Politano and Oksdath-Mansilla, 2018) using visible light and dual photosensitized electrochemical reactions (Verschueren and De Borggraeve, 2019), resulting in a more inexpensive, green, and efficient method of reaction. It took until much later for MB^+^’s staining properties to be recognized. Methylene blue belongs to the thiazine dye family and is a cationic dye. Several medical procedures involve the use of methylene blue. It possesses anti-malarial effects and has been demonstrated to be effective in the treatment of methemoglobinemia (Wainwright and Crossley, 2002; Clifton and Leikin, 2003; Tardivo et al., 2005). MB^+^ has a *τ*_f_ ∼ 1.0 ns singlet lifetime, a 664 nm absorbance, and a molar absorbance (*ε* = 94,000) (Romero et al., 2016). With a triplet lifespan of *τ*_f_ ∼ 32 μs(Pitre et al., 2016), the triplet ^3^MB^+*^ is a significantly more stable excited state (Patel et al., 2021). The photocatalytic cycles of methylene blue are depicted in Figure 1 (Patel et al., 2021). When the dye in the ground state is bombarded with visible light to produce the high-energy excited state of the dye (Dye^*^), the photoredox cycle begins. Two distinct pathways from the dye in the excited state (Dye^*^) are used to demonstrate the visible light photoredox catalysis. The Dye^*^ reductive property can be used in the presence of a sacrificial electron acceptor. In other words, as an electron donor, Dye^*^ leads to the radical cation species of Dye. Dye^*^ acts as an electron acceptor in the presence of a sacrificial electron donor (Miyabe, 2017).

Furthermore, green chemists believe that visible light irradiation is a reliable technique for environmentally friendly organic chemical syntheses since it has large energy reserves, low prices, and renewable energy sources (Mohamadpour, 2021a; Mohamadpour, 2021b).

**FIGURE 2:**
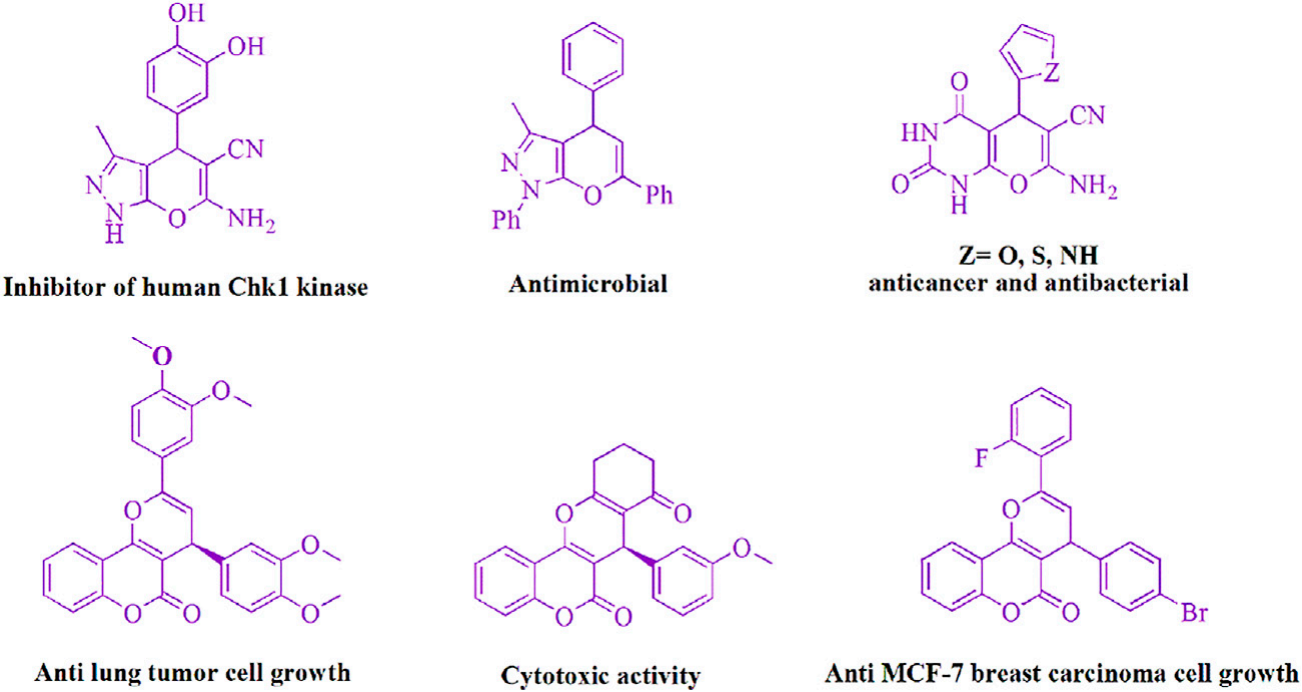
Pyran motifs can be found in a variety of medicinally important compounds.

Because of their biological and pharmacological action, the structures that makeup pyran derivatives have aroused the curiosity of biochemists and synthetic organic chemists (Figure 2) such as Chk1 kinase inhibitory activity (Foloppe et al., 2006), analgesic properties (Kuo et al., 1984), anticancer (Wang et al., 2000), vasodilatory (Ahluwalia et al., 1997), spamolytic (Ellis, 1977), antihypertensive, hepatoprotective, cardiotonic (Heber et al., 1993), vasodilator (Coates, 1990), anti-leukemic (Fokialakis et al., 2002; Beagley et al., 2003), emetic (Cannon et al., 1975), anti-anaphylactic (Biot et al., 1997), diuretic (Hafez et al., 1987), and anti-alzheimer activities (Bayer et al., 2006).

Several methods for synthesizing tetrahydrobenzo[*b*]pyran scaffolds with MCRs in the presence of various catalysts have been published. For example, CaHPO_4_ (Bodaghifard et al., 2016), SiO_2_NPs (Banerjee et al., 2011), ethylenediamine diacetate (Zhou et al., 2017), silica-bonded N-propylpiperazine sodium n-propionate (Niknam et al., 2013), I_2_ (Bhosale et al., 2007), NH_4_Al(SO_4_)_2_.12H_2_O (Mohammadi et al., 2017), NH_4_H_2_PO_4_/Al_2_O_3_ (Maleki and Sedigh Ashrafi, 2014), ACoPc-MNPs (Zolfigol et al., 2016), ZnO NPs (Banerjee and Saha, 2013), Fe_3_O_4_@SiO_2_-imid-PMA (Esmaeilpour et al., 2015), NiFe_2_O_4_@SiO_2_–H_3_PW_12_O_40_ (Maleki et al., 2016), theophylline (Mohamadpour, 2021c), triethanolamine (Rahnamaf et al., 2020), sodium alginate (Mohamadpour, 2022a), Fe_3_O_4_@SiO_2_@TiO_2_ (Khazaei et al., 2015), MgFe_2_O_4_ nanoparticles (Eshtehardian et al., 2020), trichloroisocyanuric acid (Hojati et al., 2018), Na2 eosin Y (Mohamadpour, 2021d), DABCO (Tahmassebi et al., 2011), and Pd nanoparticles (Saha and Pal, 2012). There are limitations on metal catalysts such as, expensive reagents, severe reaction conditions, monotonous yields, environmental hazards, workup processes, and long reaction times associated with these methods. A homogenous catalyst is also difficult to separate from a reaction mixture. Our goal was to investigate photocatalysts (Mohamadpour, 2021e; Mohamadpour, 2021f) in green environments in order to synthesize heterocyclic compounds that had previously been explored. This research also shows the use of MB^+^ (Mohamadpour, 2022b; Mohamadpour, 2022c) as a metal-free dye photo-redox catalyzer that is low-cost and widely available. Visible light assists Knoevenagel–Michael cyclocondensation process of aldehyde derivatives, malononitrile, and dimedone in an aqueous solvent at room temperature and in an air environment. This was a successful one-pot reaction that was completed in a timely, cost-effective, and simple manner.

## 2 Results and Discussion

**FIGURE 3:**
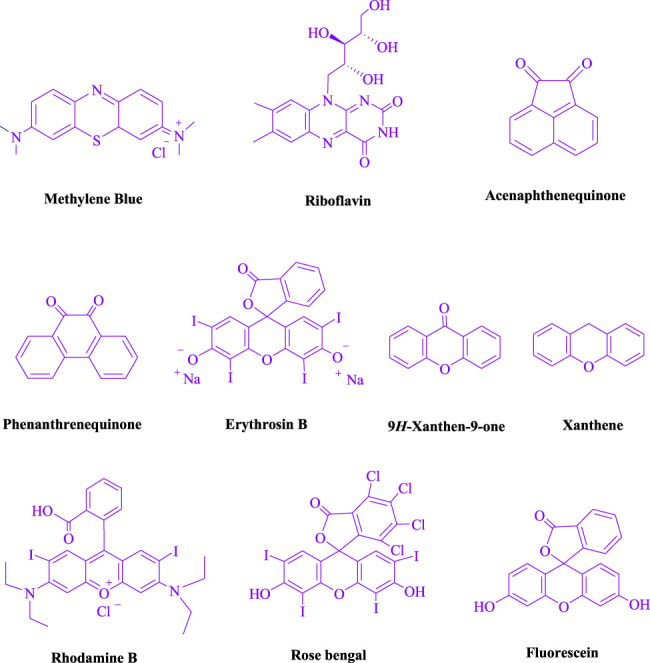
Structures of various photocatalysts.

**SCHEME 1:**
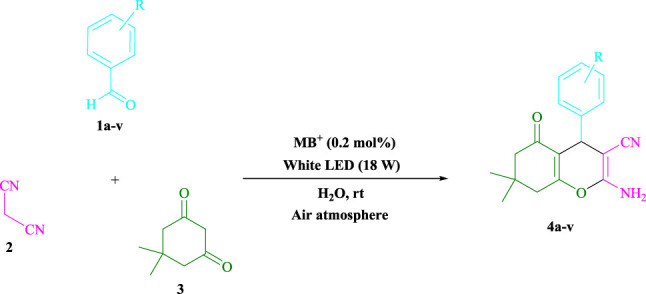
Synthesis of tetrahydrobenzo[*b*]pyran scaffolds.

To begin, LED irradiation was used to study the reaction of benzaldehyde, malononitrile, and dimedone in H_2_O (3 ml) at room temperature. In 3 ml H_2_O for 20 min, there was a 64% yield of **4a** without photocatalysts. As a way of improving the reaction, methylene blue, riboflavin, acenaphthenequinone, phenanthrenequinone, erythrosin B, 9*H*-xanthen-9-one, xanthene, rhodamine B, rose Bengal, and fluorescein (Figure 3) were examined in the same settings. This reaction proceeded with 43–97% yields and produced the acceptable matching product **4a** (Table 1). Methylene blue, according to the data, performed better in such a response. Using 0.2 mol% MB^+^, the yield was raised to 97% (Table 1, entry 3). The CH_2_Cl_2_, DMSO, toluene, THF, and DMF all resulted in decreased yields. When the reaction is carried out in EtOAc, EtOH, MeOH, H_2_O/EtOH (1:1), CH_3_CN, or solvent-free conditions, the reaction rate and yield increase. With a high yield and rate, the reaction took place in H_2_O. Using the same conditions as entry 12, a yield of 97% was obtained. In Table 2, the impact of white light on the yield was examined using a variety of light sources. Testing without the light source resulted in a small amount of **4a**. The effective synthesis of product **4a** requires visible light and MB^+^, according to the findings. Changes in the intensity of white LEDs were also used to find the improved settings (10, 12, 18, and 20 W). White LED (18 W) was found to be the best choice according to the researchers (Table 2, entry 12). Table 3 and Scheme 1 show that a wide variety of substrates were evaluated under ideal conditions. In Table 3, it appears that the benzaldehyde substituent had no influence on the outcome of the reaction. Within the reaction conditions, polar and halides were allowed. The current reaction conditions permit both electron-donating and electron-withdrawing reactions to proceed successfully. *Ortho-*, *meta-*, and *para*-substituted aromatic aldehydes have a very high yield. Various aldehydes, such as the heavier naphthaldehyde, result in a completed product with negligible yield loss. Heterocyclic aldehydes followed a similar pattern in terms of reactivity.

**TABLE 1:** Optimization of various photocatalysts^a^.

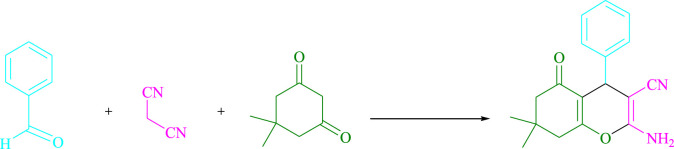				
Entry	Photocatalyst	Solvent (3 ml)	Time (min)	Isolated yields (%)
1	—	H_2_O	20	64
2	Methylene blue (0.1 mol%)	H_2_O	3	81
**3**	**Methylene blue (0.2 mol%)**	**H**_**2**_ **O**	**3**	**97**
4	Methylene blue (0.5 mol%)	H_2_O	3	97
5	Riboflavin (0.2 mol%)	H_2_O	3	61
6	Acenaphthenequinone (0.2 mol%)	H_2_O	3	46
7	Phenanthrenequinone (0.2 mol%)	H_2_O	3	43
8	Erythrosin B (0.2 mol%)	H_2_O	3	48
9	9*H*-Xanthen-9-one (0.2 mol%)	H_2_O	3	49
10	Xanthene (0.2 mol%)	H_2_O	3	47
11	Rhodamine B (0.2 mol%)	H_2_O	3	63
12	Rose bengal (0.2 mol%)	H_2_O	3	56
13	Fluorescein (0.2 mol%)	H_2_O	3	67

a Reaction conditions: malononitrile (1 mmol), benzaldehyde (1 mmol), and dimedone (1 mmol) in H_2_O, as well as a white LED (18 W) and a variety of photocatalysts, were utilized at room temperature.

**TABLE 2:** Optimization of the solvents and visible light^a^.

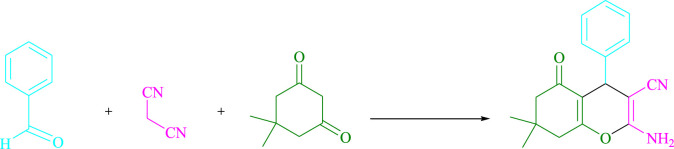				
Entry	Light source	Solvent (3 ml)	Time (min)	Isolated yields (%)
1	—	H_2_O	15	trace
2	Blue light (18 W)	H_2_O	3	90
3	Green light (18 W)	H_2_O	3	86
4	White light (10 W)	H_2_O	3	83
5	White light (12 W)	H_2_O	3	91
6	White light (20 W)	H_2_O	3	97
7	White light (18 W)	EtOAc	3	71
8	White light (18 W)	EtOH	3	76
9	White light (18 W)	—	8	74
10	White light (18 W)	MeOH	6	67
11	White light (18 W)	H_2_O/EtOH (1:1)	3	88
**12**	**White light (18 W)**	**H**_**2**_ **O**	**3**	**97**
13	White light (18 W)	CH_3_CN	3	68
14	White light (18 W)	CH_2_Cl_2_	20	33
15	White light (18 W)	DMSO	25	35
16	White light (18 W)	toluene	25	28
17	White light (18 W)	THF	15	18
18	White light (18 W)	DMF	15	24

a Reaction conditions: at room temperature, malononitrile (1 mmol), benzaldehyde (1 mmol), and dimedone (1 mmol) were added to MB^+^ (0.2 mol %).

**TABLE 3:** Synthesis of tetrahydrobenzo[*b*]pyran scaffolds.

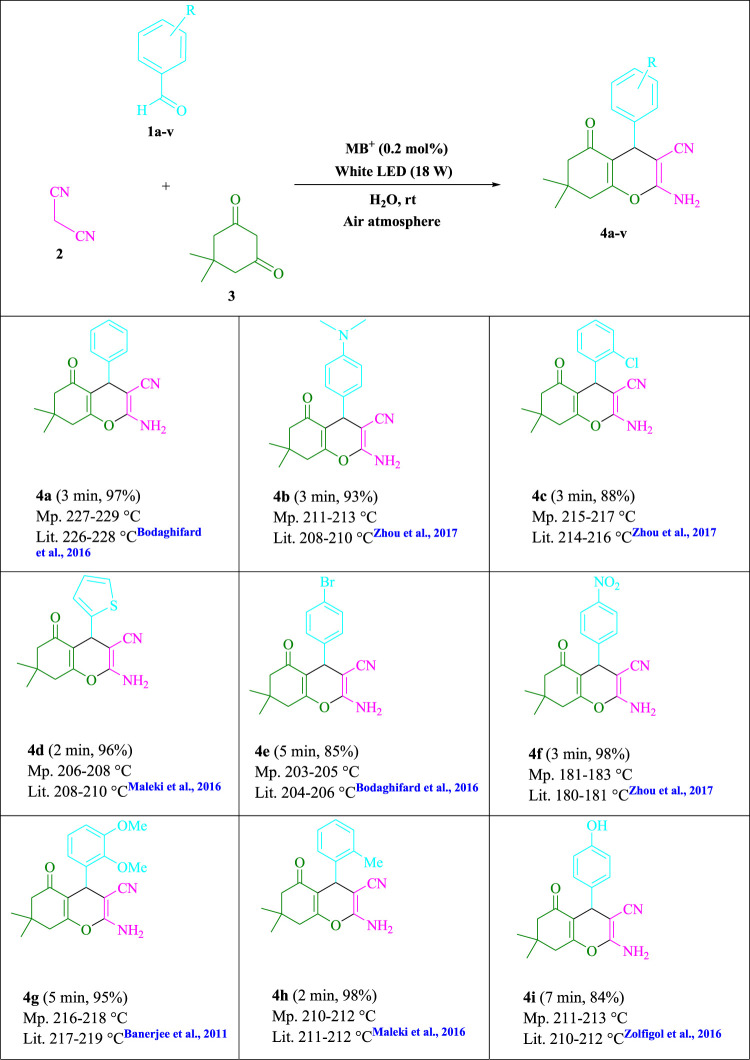
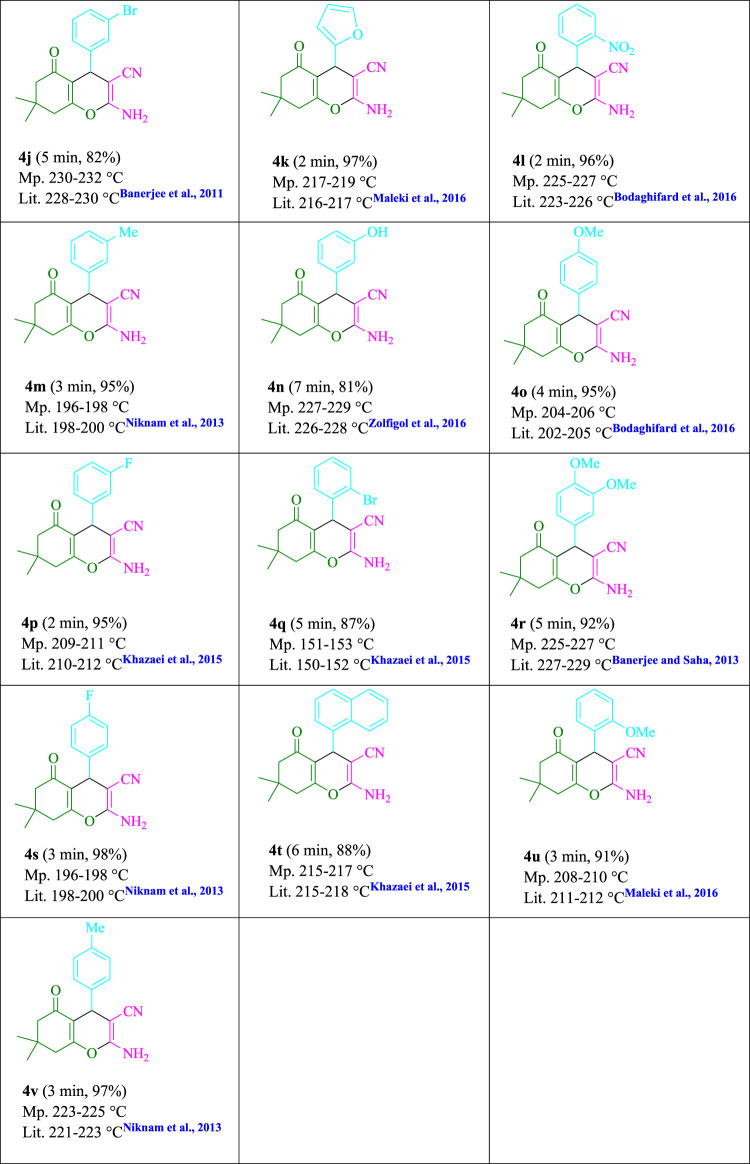

Likewise, Table 4 displays the turnover number (TON) and turnover frequency (TOF). A higher TON and TOF numerical value mean less catalyst is utilized, and a higher yield, and the catalyst becomes more efficient with increasing value.

**TABLE 4:** Calculated turnover number (TON) and turnover frequency (TOF).

Entry	Product	TON	TOF	Entry	Product	TON	TOF
1	**4a**	485	161.6	12	**4l**	480	240
2	**4b**	465	155	13	**4m**	475	158.3
3	**4c**	440	146.6	14	**4n**	405	57.8
4	**4d**	480	240	15	**4o**	475	118.7
5	**4e**	425	85	16	**4p**	475	237.5
6	**4f**	490	163.3	17	**4q**	435	87
7	**4g**	475	95	18	**4r**	460	92
8	**4h**	490	245	19	**4s**	490	163.3
9	**4i**	420	60	20	**4t**	440	73.3
10	**4j**	410	82	21	**4u**	455	151.6
11	**4k**	485	242.5	22	**4v**	485	161.6

**SCHEME 2:**
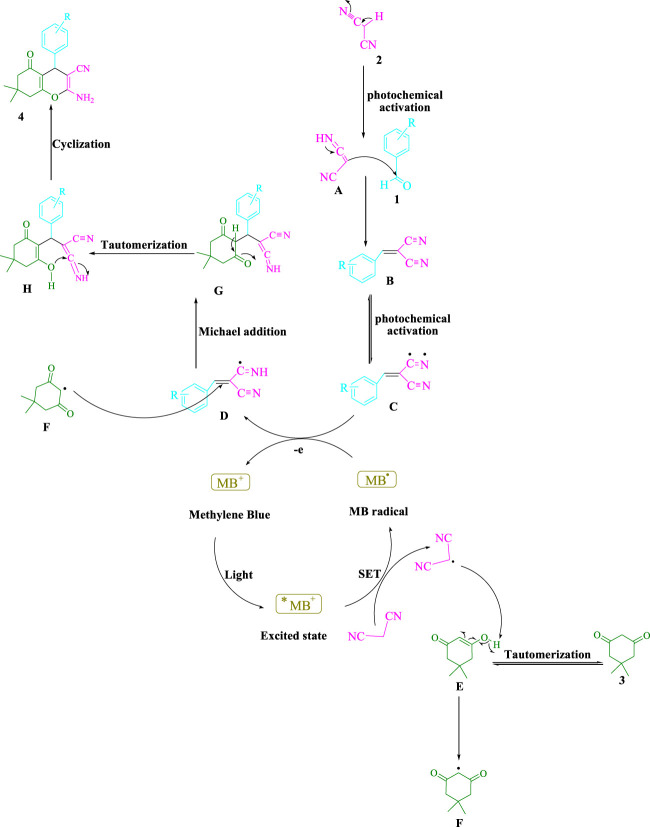
There has been a mechanistic approach presented for synthesizing tetrahydrobenzo[*b*]pyran scaffolds.

The chosen strategy is depicted in Scheme 2. It is possible to tautomerize malononitrile (**2**) by exposing it to visible light (**A**). After that, the aldehydes (**1**) and (**A**) are joined to generate arylidenemalononitrile (**B**), which is photochemically activated to yield a radical intermediate (**C**). More energy can be utilized to accelerate this reaction, altering visible light. According to recent studies (Patel et al., 2021), visible light energy is utilized by this widely available cationic dye to create catalytic approaches that use single-electron transfer (SET) as well as energy transfer (EnT). To boost the visible-light–induced ^*^MB^+^, a SET approach is used to produce the malononitrile radical. The energy transfer (EnT) activity between the radical adduct (**C**) and the MB radical produces the intermediate (**D**) and ground-state MB. The intermediate (**F**) is formed when the malononitrile radical takes a hydrogen atom from (**E**). The intermediates (**F**) and (**D**) combine as a Michael acceptor to generate (**G**), which then undergo intramolecular cyclization and tautomerization to give rise to the final product (**4**).

A comparison of the catalytic ability of several catalysts described in the literature is presented in Table 5 for the synthesis of tetrahydrobenzo[*b*]pyran scaffolds. In the presence of visible light, it could possess a number of useful properties, such as the need for a small amount of photocatalyst, a rapid reaction time, aqueous solvents, and the absence of byproducts. The atom–economic protocol is exceedingly successful at multigram scales and has significant industrial implications. Their efficiency and purity set them apart from other materials.

**TABLE 5:** Comparing the catalytic characteristics of different catalysts described in the text for the production of catalyst **4a** ^a^.

Entry	Catalyst	Conditions	Time/yield (%)	Reference
1	CaHPO_4_	H_2_O/EtOH, 80°C	120 min/91	Bodaghifard et al. (2016)
2	SiO_2_ NPs	EtOH, rt	25 min/94	Banerjee et al. (2011)
3	Silica-bonded N-propylpiperazine sodium n-propionate	H_2_O/EtOH, Reflux	25 min/90	Niknam et al. (2013)
4	I_2_	DMSO, 120°C	3.2 h/92	Bhosale et al. (2007)
5	NH_4_Al(SO_4_)_2_.12H_2_O	EtOH, 80°C	120 min/92	Mohammadi et al. (2017)
6	NH_4_H_2_PO_4_/Al_2_O_3_	EtOH, Reflux	15 min/86	Maleki and Ashrafi, (2014)
7	Fe_3_O_4_@SiO_2_-imid-PMA	H_2_O, Reflux	20 min/94	Esmaeilpour et al. (2015)
8	Theophylline	H_2_O/EtOH, rt	10 min/89	Mohamadpour, (2021c)
9	Trichloroisocyanuric acid	EtOH, 80 °C	10 min/90	Rahnamaf et al. (2020)
**10**	**MB** ^**+**^	**visible light irradiation, H**_**2**_ **O, rt**	**3 min/97**	**This work**

a Based on the benzaldehyde, malononitrile, and dimedone three-component synthesis.

## 3 Experiment

### 3.1 General

A 9100 electro–thermal apparatus was used to determine the melting points of all compounds. A Bruker (DRX-400 and DRX-300) instrument was also used to record the nuclear magnetic resonance (1HNMR) spectra using CDCl3 as the solvent.

#### 3.1.1 Preparation of Tetrahydrobenzo[b]pyran Scaffolds in General (4a–v)

Methylene blue (0.2 mol%) was mixed with dimedone (**3**, 1.0 mmol), malononitrile (**2**, 1.0 mmol), and aldehydes (**1**, 1.0 mmol) in H_2_O (3 ml) and agitated at room temperature under white LED (18 W) irradiation. The reaction, which used *n*-hexane/ethyl acetate (3:1) as the eluent, was monitored using TLC. As a result of the reaction, the resultant substance was screened and rinsed with water, and the crude solid was crystallized from ethanol in order to yield the pure chemical without further purification. If we could make the aforementioned compounds using gram scale methods, we would be able to scale up to the level of pharmaceutical process development. 50 mmol of m-tolualdehyde, malononitrile, and dimedone were used in one experiment. The large-scale reaction ran well, requiring only 3 min to complete, and the product was recovered using typical filtration processes. The ^1^HNMR spectrum of this material suggests that it is spectroscopically pure. After comparing the spectroscopic data, the products were categorized (^1^HNMR). The ^1^HNMR spectra files are provided in the Supplementary Material.

## 4 Conclusion

According to the findings, using a single-electron transfer (SET)/energy transfer (EnT), a radical tandem Knoevenagel–Michael cyclocondensation process of aldehyde derivatives, malononitrile, and dimedone can be used to generate metal-free tetrahydrobenzo[*b*]pyran scaffolds. In an aqueous solution and an air atmosphere at room temperature, visible light is used as a renewable energy source. Green protocol advantages include the use of minimal amounts of photocatalyst, excellent yields, a reaction side that is highly efficient, safe conditions for the reaction, and a speedy procedure without the use of toxic chemicals or solvents. The purification process did not require chromatography. A model substrate reaction at the multigram scale demonstrates that this reaction can be scaled up without compromising the outcome. Due to these advantages, this technology offers significant benefits for industrial applications and for environmental concerns.

## Data Availability

The datasets presented in this study can be found in online repositories. The names of the repository/repositories and accession number(s) can be found in the article/Supplementary Material.
