# Supplementary material for: Methylene Blue as a Photo-Redox Catalyst: The Development Synthesis of Tetrahydrobenzo[b]pyran Scaffolds via a Single-Electron Transfer/Energy Transfer
Source: Front Chem. 2022 Jul 18;10:934781. doi: 10.3389/fchem.2022.934781 (PMC9339953; doi:10.3389/fchem.2022.934781)
Supplement: Supplementary file 1 [file DataSheet1.pdf]

Supporting Information

Methylene blue as a photo-redox catalyst: the development synthesis of tetrahydrobenzo[*b*]pyran scaffolds *via* a single-electron transfer/energy transfer

Farzaneh Mohamadpour *

School of Engineering, Apadana Institute of Higher Education, Shiraz, Iran

* *Corresponding author. mohamadpour.f.7@gmail.com*

2-Amino-4-(2,3-dimethoxyphenyl)-7,7-dimethyl-5-oxo-5,6,7,8-tetrahydro-4Hchromene-3-carbonitrile (4g)

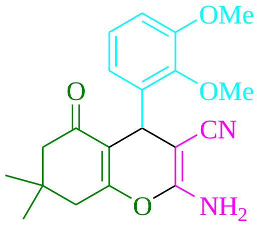

Yield: 95%; M.p. 216-218 °C; ^{1}HNMR (300MHz, $CDCl_3$): δ 1.10 (3H, s, CH_3), 1.14 (3H, s, CH_3), 2.25 (2H, s, CH_2), 2.47 (2H, s, CH_2), 3.77 (3H, s, OCH_3), 3.83 (3H, s, OCH_3), 4.47 (2H, s, NH_2), 4.73 (1H, s, CHAr), 6.68-6.84 (3H, m, ArH).

2-Amino-4-(3-methylphenyl)-7,7-dimethyl-5-oxo-5,6,7,8-tetrahydro-4Hchromene-3-carbonitrile (4m)

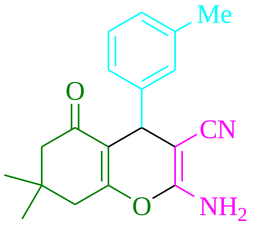

Yield: 95%; M.p. 196-198 °C; ^{1}HNMR (400MHz, $CDCl_3$): δ 1.06 (3H, s, CH_3), 1.13 (3H, s, CH_3), 2.23 (2H, d, *J*=5.6 Hz, CH_2), 2.31 (3H, s, CH_3), 2.46 (2H, s, CH_2), 4.38 (1H, s, CHAr), 4.52 (2H, s, NH_2), 7.09-7.15 (3H, m, ArH), 7.28 (1H, s, ArH).

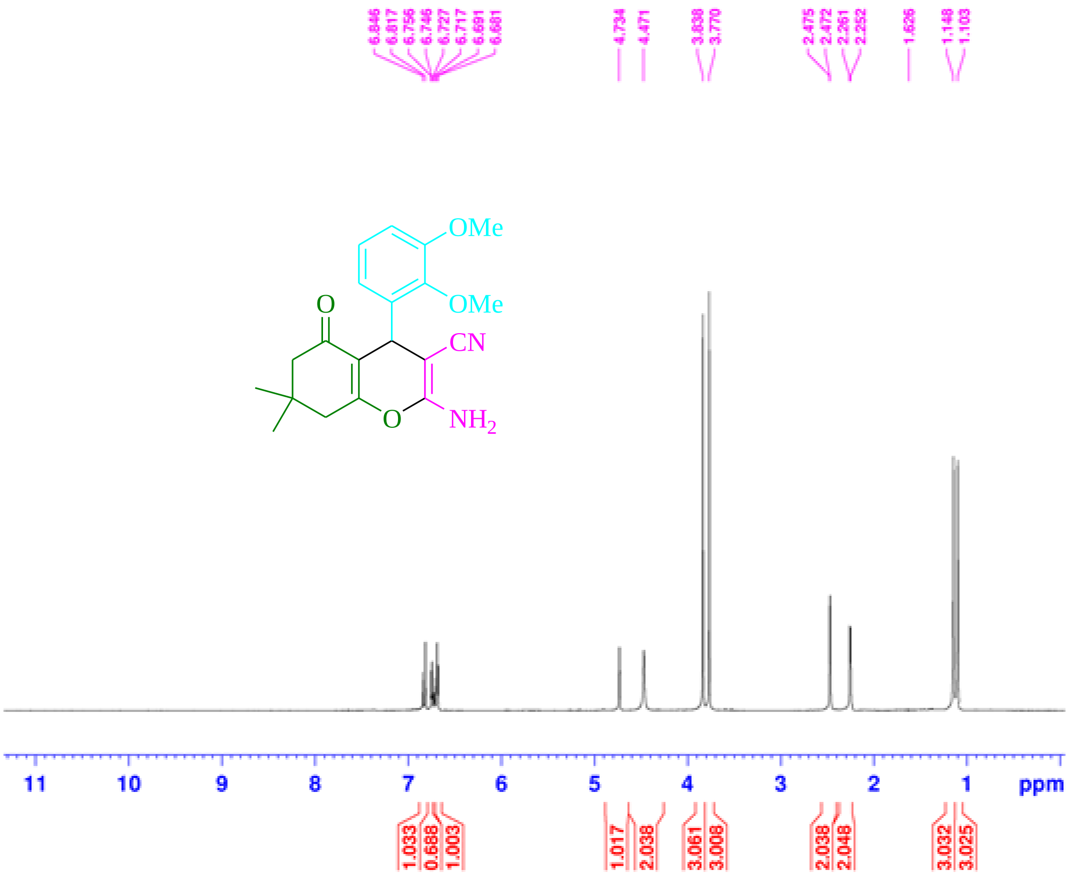

Figure 1. ^{1}HNMR Spectrum of compound (300 MHz, $CDCl_3$) of **4g**

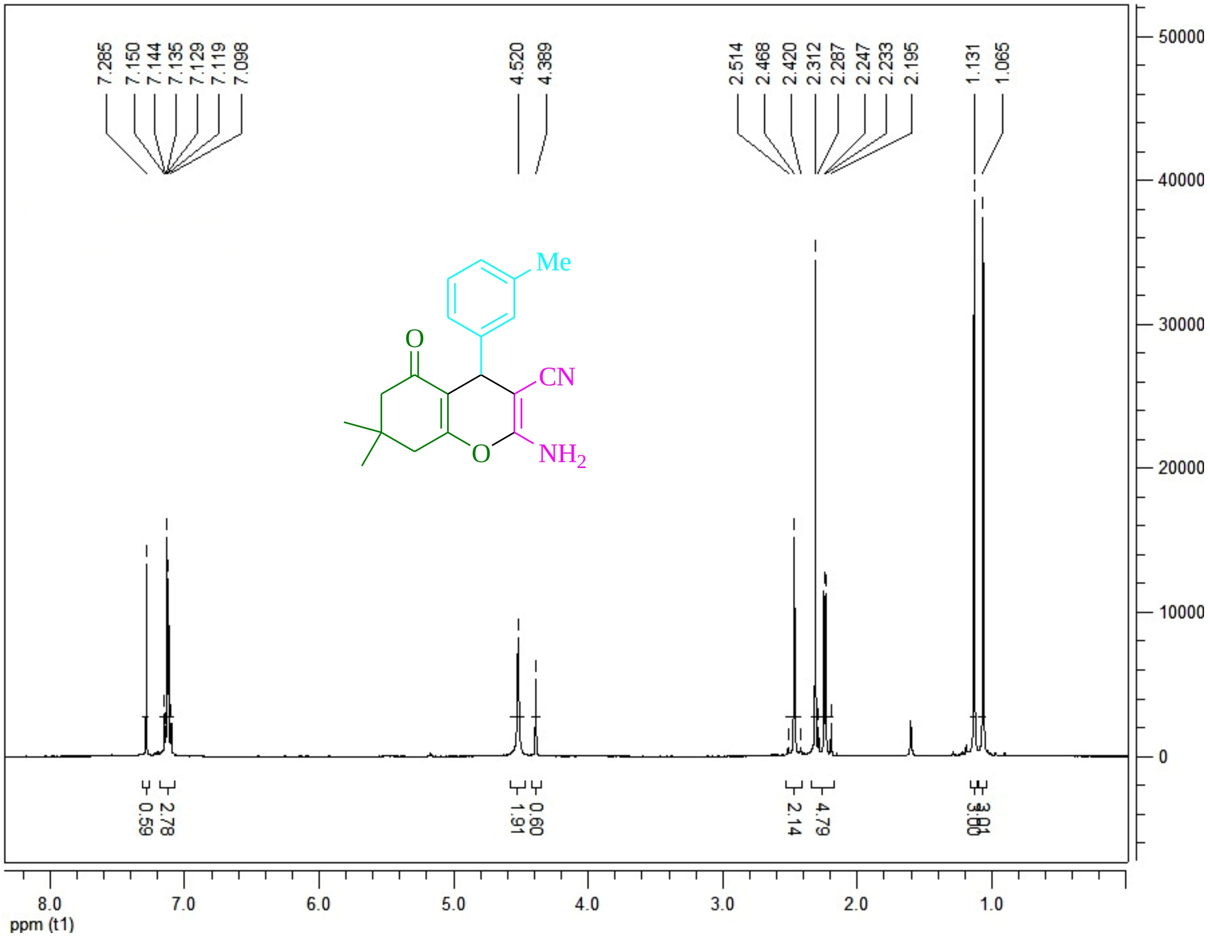

Figure 2.^{1}HNMR Spectrum of compound (400 MHz, $CDCl_3$) of **4m**
